# Longitudinal association of health behaviors and health-related quality of life with military spouse readiness

**DOI:** 10.1186/s12889-024-18786-2

**Published:** 2024-05-18

**Authors:** Nida H. Corry, Sharmini Radakrishnan, Christianna S. Williams, Kelly A. Woodall, Valerie A. Stander

**Affiliations:** 1 Health Care Evaluation, NORC at the University of Chicago, Chicago, IL USA; 2 Division of Health and Environment, Abt Associates, Rockville, MD USA; 3 Leidos, Reston, VA USA; 4 Deployment Health Research Department, Naval Health Research Center, San Diego, CA USA; 5 10 Fawcett St, Cambridge, MA 02138 USA

**Keywords:** Health behaviors, Military families, Health-related quality of life

## Abstract

**Background:**

Unhealthy behaviors impose costs on health-related quality of life (HRQOL) reducing productivity and readiness among military members (Hoge et al., JAMA 295:1023–32, 2006; Mansfield et al. 362:101–9, 2010). Among married personnel in particular, patterns of spouse health behaviors may play an interdependent role. As a result, the identification of military spouse health factors related to readiness may inform strategies to screen for and identify those in need of greater support and enhance readiness. This study explored behavioral and HRQOL predictors and potential mediators of military spouse readiness utilizing data from the Millennium Cohort Family Study.

**Methods:**

The analytic sample comprised of 3257 spouses of active-duty, non-separated service members who responded to both waves 1 and 2 of the survey. Sample characteristics are described with respect to demographics (e.g., age, sex, race/ethnicity, etc.), readiness measures (i.e., military satisfaction, lost workdays, health care utilization, military-related stress, and satisfaction), health behaviors (i.e., exercise, sleep, smoking, and alcohol use) and HRQOL (Veterans RAND 12-Item Short Form Survey). We conducted multivariate mediation analyses to evaluate the role of mental and physical HRQOL as mediators between the baseline health behaviors and the health readiness outcomes at follow-up, while adjusting for spouse and service member demographics.

**Results:**

HRQOL had direct effects for all five readiness outcomes examined. Multiple health behaviors (insomnia, smoking, binge drinking, and exercise) were further significantly associated with spouse readiness outcomes, although most effects were mediated through HRQOL, suggesting this may be a useful index of military spouse readiness. Insomnia was the specific health behavior most consistently associated with poorer readiness across outcomes, and effects were only partially mediated by physical and mental HRQOL.

**Conclusions:**

The results show spouse health behaviors are directly and indirectly (through HRQOL) associated with readiness indicators. This suggests that assessments of modifiable health behaviors (e.g., insomnia symptoms) and mental and physical HRQOL are important indicators of readiness among military spouses and should be used to inform future programs designed to improve population health.

## Background

Military force readiness and retention relies not only on the adjustment and satisfaction of the service member, but also on the readiness of their family which is heavily influenced by economic well-being and quality of life dimensions [1–4]. Military spouse readiness, including providing support to the service member for continued service, is an essential component of military readiness and can affect service member retention and performance [5–7], as well as family outcomes [8, 9]. Military spouse readiness is defined as “the state of being prepared to effectively navigate the challenges of daily living experienced in the unique context of military service, to include: mobility and financial readiness, mobilization and deployment readiness, and personal and family life readiness” [10]. Spouse readiness is multi-faceted and thus can be operationalized in several ways.

### Spouse readiness measures

Spouse readiness is associated with spouses’ satisfaction with military life. Among deployed service members in Southwest Asia, family-related stress significantly affected married service members’ ability to concentrate or do their job [11]. In addition, spouse dissatisfaction with military support and the perception that military life was incompatible with family life were the two largest predictors of service members’ intentions to leave service following deployment to Operation Desert Storm [12]. Higher levels of spouse satisfaction with the military are also associated with improved psychological well-being for the spouse and active-duty service member [13]. Using data from DoD-sponsored epidemiologic surveys [14], spouses’ support for service members’ military careers has been shown to be critical to retention; over a two-year period, 93% of personnel remained in service when spouses supported their military careers, while only 44% remained without spousal support. Additionally, among military spouses in the Defense Health Agency (DHA sponsored Millennium Cohort Family Study, work-family conflict and both spouse and service member military satisfaction were important mediators in predicting the impact of military and family factors on voluntary service separation [15].

The stress level spouses experience during challenging military-related experiences, such as deployment separation, family financial stress, and increased periods of operational tempo, are important metrics of spouse readiness. A study of Army spouses reported that service member deployments were a stressor [16], with at least half of spouses reporting feeling stressed and overwhelmed during a partner’s deployment [17]. Spouses with high deployment-related stress have elevated risks for poor physical health [16–18], psychological distress [8], and challenges balancing new responsibilities. Among spouses of deployed service members, use of mental health services increased 20% during their first deployment and remained elevated between the first and second deployment due to increased stress [19]. Financial stress related to struggling to make ends meet, ineffectively managing finances, or the perception of financial stress can influence aspects of military family life such as well-being, relationships, and parenting [4]. Regarding frequent relocations, military families find it difficult to constantly readjust and readapt to new places [20]. When spouses can cope with military-life challenges without high levels of stress, families are better prepared to support the service member in their military responsibilities.

For many military spouses, employment is an essential aspect of quality of life for personal reasons as well as bridging financial gaps in their family with a secondary income. If a spouse has a health condition or injury leading to lost workdays, the resulting financial burden can adversely affect spouse readiness. The cost of lost workdays specifically for military spouses is unknown; however, research on the general US population is relevant, because most employed military spouses are in the civilian workforce. One study of US workers found employers lost $226 billion in 2002 due to lost productivity time related to employees work absence for personal health (0.54 h per week) and work absence for family health (0.12 h per week) [21]. This study also observed women reported 30% more lost productivity time compared with men.

### Factors associated with spouse readiness

Although research to date on spouse readiness is limited, compared to research focused on service member readiness, findings suggest that several health behaviors and quality of life dimensions may influence spouse readiness. For instance, research on service members has demonstrated that sleep significantly affects military readiness [22]. An individual’s quality and quantity of sleep may impact cognitive processing skills [23] and performance, thus affecting readiness [24, 25]. Furthermore, poor sleep is associated with HRQOL [26] and various health outcomes [27, 28] that could indirectly impact readiness. However, it is not clear how the sleep quality of military spouses may affect spouse readiness.

Tobacco use and high alcohol consumption increase the risk of chronic diseases and healthcare utilization and costs the DoD $989 million per year for related medical care [29]. According to the most recent DoD Health-Related Behaviors Survey of active-duty personnel, one in three are current binge drinkers and approximately 13% of AD service members reported current smoking or current use of smokeless tobacco [30]. Among military spouses in the Millennium Cohort Family Study, the majority met most Healthy People 2020 goals [31] including sleep, alcohol use, and tobacco use [32]; but still, substantial numbers did not (e.g., 39% did not achieve targets for sleep). Further, the concordance among military spouses and service members was relatively high for alcohol use (54%) and tobacco use (60%), although fewer service members met these goals compared to spouses [32]. Assessing the impacts of spousal alcohol and tobacco use on spouse readiness may be elucidating to inform prevention and treatment resources.

A common conceptualization for health-related quality of life (HRQOL) is the emotional and physical capacity of an individual to function in their daily lives [33]. Overarching HRQOL may be impacted both positively and negatively by an individual’s specific health behaviors or by certain health conditions. For example, a study of Army soldiers found that an item measuring self-rated general health was associated with sleep quality, emotional fitness, and Army Physical Fitness Test (APFT) scores [34]. While there is evidence that each of the aforementioned health behaviors impact healthcare costs and functional outcomes, it is less well known how specific behaviors in conjunction with or mediated through spousal HRQOL are associated with spouse readiness. The DHA already globally assesses HRQOL among care-seeking beneficiaries (e.g., https://health.mil/Military-Health-Topics/Access-Cost-Quality-and-Safety/Health-Care-Program-Evaluation/MHS-Patient-Satisfaction-Surveys) and the DHA has expressed interest in more systematic monitoring of HRQOL as a bellwether for readiness. Achieving a more nuanced understanding of HRQOL and how that relates to military population readiness, as well as how effective HRQOL is as an indicator would enable those efforts to better address and promote readiness.

### Study aims

Military spouses experience unique military-related stressors, including deployment separation, financial stress, and increased periods of operational tempo, that may ultimately influence familial support for the service member’s military responsibilities. Unhealthy behaviors impose healthcare costs and productivity loss that reduce readiness; in contrast, positive lifestyles produce savings for the military health system [35, 36]. In providing comprehensive healthcare to the military community, the DHA strives to assess and target modifiable health behaviors among beneficiary families, to support both individual and population-level health and readiness. Therefore, identification of spousal health factors associated with spouse readiness within military populations can inform strategies to improve quality of care, health, and readiness among service members and their families.

This study sought to identify “bellwether” indicators for the health-related drivers of military readiness to inform DHA’s health screening and referral practices. This study assessed behavioral and quality of life factors as indicators of military spouse readiness utilizing data from the Millennium Cohort Family Study. Note that when we refer to spouses in this study, we are referring to the Family Study participant. We sought to assess the longitudinal relationship between health factors (i.e., HRQOL, physical activity, sleep difficulties, and alcohol and tobacco use) and readiness (i.e., military satisfaction, lost workdays, health care utilization, military-related stress) among military spouses of active-duty service members. We examined HRQOL as a mediator that facilitates the relationship between specific health behaviors and spouse readiness outcomes, and assessed if HRQOL could effectively be used as a gross screener for health-related factors associated with readiness.

## Methods

### Sample design and study participants

This study used data from waves 1 (2011–2013) and 2 (2014–2016) of the Millennium Cohort Family Study (Family Study) to assess the longitudinal relationship between health factors (HRQOL, exercise, sleep, and alcohol use) and spouse readiness. The Family Study baseline sample consists of 9,872 service member/spouse dyads. The service members are participants of the Millennium Cohort Study who were married and had 2 to 5 years of military service at the time they were recruited into the study. Spouses of participating service members were then recruited to participate in the Family Study. The Family Study methods are described in more detail elsewhere [37]. This work was approved by the Naval Health Research Center Institutional Review Board in compliance with all applicable Federal regulations governing the protection of human subjects, to include obtaining a signed informed consent form for each participant. Research data were derived from an approved Naval Health Research Center Institutional Review Board protocol, number NHRC.2015.0019.

**Fig. 1:**
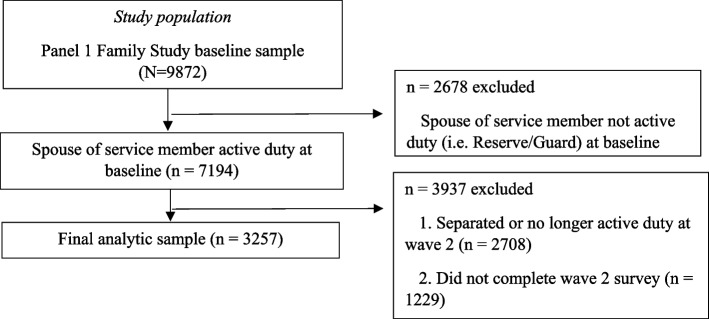
Sample selection

To analyze spouse readiness, we limited the sample to spouses of active-duty, non-separated service members who responded to both waves 1 and 2 of the survey. The final analytic sample comprised 3,257 spouses. Figure 1 below shows the sources of sample loss.

### Measures

We assessed spouse readiness using five outcome measures. Three outcomes were constructed from wave 2 of the Family Study survey and two were from Military Health System Data Repository (MDR) spouse medical records: 1) lost workdays (survey), 2) inpatient days (MDR), 3) outpatient days (MDR), 4) satisfaction with support from the military (survey), and 5) military-related stress (survey).

*Lost workdays* were measured as the number of self-reported days over the past 3 years the spouse was unable to work or perform their usual activities due to illness or injury (excluding lost time for pregnancy and childbirth) (min = 0, max = 570).

*Inpatient days* per year was computed as the number of inpatient hospitalization days reported in MDR that occurred between the spouse’s wave 1 and wave 2 interviews, divided by the number of years between wave 1 and wave 2 (min = 0, max = 41.3). *Outpatient days* per year was similarly computed as the number of outpatient medical visit days per year in MDR between waves 1 and 2, divided by the number of years between waves 1 and 2 (min = 0, max = 133.8). The number of years between waves was computed as the number of days between the wave 1 and wave 2 interviews, divided by 365.25. If someone had multiple outpatient visits in one day, it was counted as just one outpatient visit day.

*Satisfaction with perceived military support* was computed by taking the mean of two self-reported items that assessed satisfaction with the military’s efforts to help 1) the service member, and 2) the spouse and their family with the stresses of military life. Response options for each item range from “poor” to “excellent,” and are coded from 0–4.

*Military-related stress* was computed as the mean of five self-reported items measuring the spouse’s stress response to any combat deployments, non-combat deployments, intensified training schedules, permanent changes of station, or family financial stress experienced. The response options for each item ranged from “not all stressful” to “very stressful,” coded from 0–3. For each spouse, responses for all experiences occurring in the last 12 months were averaged. This outcome measure was intended to capture the respondent’s stress response rather than stress exposure, and the total stressors experienced was included as a covariate in the model.

*Health behaviors* assessed as predictors of readiness on the survey at wave 1 included: exercise, sleep, smoking, and alcohol use. Exercise was measured using the number of minutes in a typical week that the spouse engaged in moderate or vigorous-intensity exercise. This measure was computed as the number of moderate-intensity exercise minutes per week added to two times the number of vigorous-intensity exercise minutes per week [31], with scores ranging from 0 to 5000. Note that the original minutes of physical activity variable had a maximum of 11,515 min. After reviewing the variable distribution, we truncated the variable at 5000 min (99th percentile in the distribution) for use in analysis, so that all spouses with minutes greater than 5000 were assigned this new maximum value of 5000. The Insomnia Severity Index (ISI) was administered to assess sleep problems, specifically the nature, severity and impact of insomnia as a behavioral health condition, as it is the most commonly reported sleep problem in the general population [38]. The Insomnia Severity Index (ISI) score [39] includes seven items, each scored on a 0 to 4 Likert scale. Scores ranged from 0–28, with a higher score indicating more insomnia symptoms.

We assessed smoking using an item from the Family Study which asked the spouse “How many cigarette packs per day did you or do you smoke?” Spouses who never smoked or did not smoke in the past year were assigned 0 for this variable, and response options ranged from 0 (never smoked in the past year) to 4 (more than 2 packs per day). Finally, we assessed alcohol use using two items on binge drinking frequency. Male spouses were asked “How often did you typically have 5 or more drinks of alcoholic beverages within 2-h period?” Female spouses were asked how often they had 4 or more drinks in 2 h [40]. Both items had response options ranging from “never” to “more than 4 times per month,” coded as 1–4. Spouses who never used alcohol or did not drink in the past year were coded as 1 (“never”). We used all four health behavior measures as continuous predictors in the model.

*Health-related quality of life measures* were assessed on the survey at wave 1 using the Veterans RAND 12-Item Short Form Survey (SF-12) [41], a standardized and widely used brief assessment of health-related quality of life, which captures eight health dimensions: physical functioning, physical health problems, bodily pain, general health, vitality, social functioning, psychological distress, and psychological well-being, by asking about the self-reported effect of physical and mental health problems on basic components of day-to-day functioning [42–44]. Each of the 12 questions in this assessment is weighted differently to yield the mental component score (MCS) and physical component score (PCS). MCS and PCS scores have normative values with a mean of 50 and standard deviation of 10 to compare to other US populations, with higher scores reflecting a more favorable health status [45–49]. We assessed MCS and PCS continuously for this study, with total scores ranging from -3 to 71 (MCS) and 12 to 70 (PCS).

*Covariates* assessed in addition to the independent variables described above, included spouse socio-demographics and service member military characteristics as covariates in the model. The spouse characteristics included gender, age, race and ethnicity, educational attainment, marital status, number of children, and the spouse’s prior or current military service. Service member characteristics included military paygrade and service branch. For the military-related stress outcome only, we included the number of stressors experienced as an additional covariate. Finally, we also included time between the wave 1 and 2 interviews (in years) as a covariate in all models.

### Analyses

We generated descriptive statistics on the spouse demographics as well as the readiness measures, health behaviors, and HRQOL measures, and reported weighted means and unweighted sample sizes. Next, we estimated unadjusted bivariate regression models to assess the correlation between the predictors and spouse readiness. Finally, we then estimated multivariate mediation models assessing the direct and indirect effects of health behaviors on readiness, with mental and physical HRQOL as mediators. We used Mplus version 8 software for the mediation modeling and SAS version 9.7 for the descriptive statistics [50, 51]. We used the indirect effects approach suggested by Preacher and Hayes, which focused on an investigation of indirect effects and associated confidence intervals with robust standard errors estimation. Each outcome was modeled separately, resulting in five separate mediation models.

**Fig. 2:**
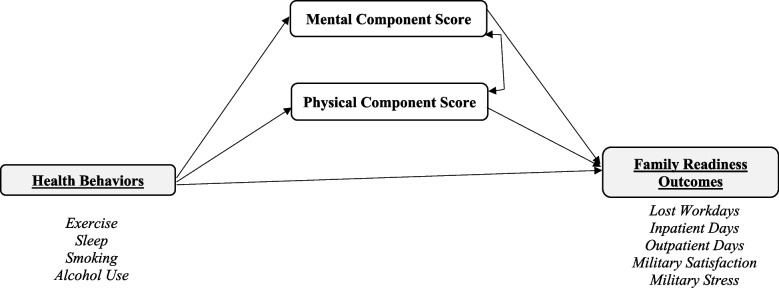
Conceptual mediation model of family readiness. Note. Each outcome was modeled separately, resulting in five separate mediation models. Additionally, each model adjusted for covariates, including spouse’s sex, age, race/ethnicity, marital status, education, number of children, military experience, time between baseline and follow-up surveys, service member’s pay grade, and service branch

The Family Study survey weights were used with all models to account for sampling design and nonresponse bias [52]. We standardized all continuous predictors – including the health behaviors and HRQOL measures—as well as the military satisfaction and military stress outcomes before estimating the models. Figure 2 illustrates our conceptual model of the relationships between health behaviors, HRQOL, and spouse readiness. In this model, the direct effect of health behaviors on readiness is their unique effect independent of the mediator (HRQOL), as illustrated by the arrow running directly from health behaviors to the readiness outcomes. The indirect effect of health behaviors on readiness is their effect on these outcomes that works through mediator, as illustrated by the arrows running from health behaviors to the HRQOL measures (Mental and Physical Component Scores), and the arrows from HRQOL to readiness. The total effect of health behaviors on readiness is the sum of their direct and indirect effects.

## Results

### Population description

Table 1 shows descriptive statistics – both weighted means/frequencies and unweighted sample sizes – for the spouse demographics, readiness outcomes, health behaviors, and HRQOL measures. Most spouses were female (89.7%), between 25 and 34 (65.4%) years of age, and non-Hispanic White (67.6%). Most had some college education or an associate degree. The majority of spouses were married (98.9%) and had no prior military service (82.9%). Most spouses had least one child (65%). Most spouses’ service member partners served in the Army (38.4%), with the remainder serving in the Air Force (38.4%), Navy (19%), Marine Corps (13.8%), and Coast Guard (4.9%). The average time between the spouses’ baseline and follow up interviews was 2.2 years.

**Table 1:** Descriptive Statistics

Variable	Unweighted N^a^ (*N* = 3257)	Weighted %
Spouse Readiness Outcomes (Wave 2)		
**Lost workdays in past 3 years**		
0	1492	52.96
1–7	760	25.38
8–14	236	9.80
15–570	347	11.86
Mean (SE)	14.86 (1.53)	
**Inpatient days per year**		
0	1858	60.95
0.1–2	1257	34.19
2.001–3	72	3.01
3 +	70	1.85
Mean (SE)	0.56 (0.03)	
**Outpatient days per year**		
0	43	1.49
0.1–3	570	17.75
3.001–5	503	15.66
5.0001–10	1104	31.28
10.0001–15	515	16.31
15 +	522	17.50
Mean (SE)	9.48 (0.23)	
**Satisfaction with military support**		
0	287	13.56
0.1–1	626	22.93
1.1–2	883	31.12
2.1–3	581	21.27
3.1–4	265	11.11
Mean (SE)	1.82 (0.04)	
**Military stress**		
0–0.9	345	16.98
1–1.9	989	44.15
2–2.9	686	31.26
3	182	7.60
Mean (SE)	1.49 (0.03)	
Health behaviors (Wave 1)		
**Moderate/vigorous exercise minutes**		
0 min/wk	292	12.21
1–120 min/wk	557	17.22
121–240 min/wk	618	17.88
241–360 min/wk	510	15.41
360–480 min/wk	373	11.30
481–5000 min/wk	794	25.98
Mean (SE)	403.42 (15.40)	
**Insomnia Severity Index (ISI) score**		
No clinically sig. insomnia (0–7)	2004	58.87
Subthreshold insomnia (8–14)	870	28.52
Clinical insomnia (moderate severity) (15–21)	289	10.04
Clinical insomnia (severe) (22–28)	67	2.57
Mean (SE)	7.17 (0.17)	
**Cigarette packs per day**^b^		
Never smoked/did not smoke in past year (0)	2834	82.36
Less than half pack per day (1)	217	9.77
Half to 1 pack per day (2)	150	6.37
1 to 2 packs per day (3)	29	1.44
More than 2 packs per day (4)	1	0.06
Mean (SE)	0.27 (0.02)	
**Binge drinking frequency in past year**^b^		
Never (1)	2645	79.98
Monthly or less (2)	513	17.51
2–4 times/month (3)	43	1.55
> 4 times/month (4)	27	0.96
Mean (SE)	1.23 (0.01)	
Health-related Quality of Life (HRQoL) measures		
**Transformed mental score (MCS) 12**		
-3–25	97	3.96
25.001–50	1300	41.61
50.001–60	1690	48.90
60.001–71	153	5.53
Mean (SE)	49.18 (9.61)	
**Transformed physical score (PCS) 12**		
12–50	626	23.02
50.001–60	2368	70.05
60.001–70	248	6.93
Mean (SE)	53.65 (7.07)	
Spouse Characteristics		
**Spouse gender**		
Male	261	10.33
Female	2996	89.67
**Spouse age**		
17–24 years	606	25.57
25–34 years	2339	65.41
35 + years	312	9.01
**Spouse race/ethnicity**		
White non-Hispanic	2560	67.64
Black non-Hispanic	120	8.99
Hispanic	303	13.25
Other	260	10.12
**Spouse education**		
HS Degree or less	292	16.30
Some college/Associates degree	1308	52.25
Bachelors degree or higher	1653	31.45
**Marital status**		
Married	3236	98.92
Divorced/separated/widowed	21	1.08
**Number of children**		
0	1221	35.00
1	924	28.41
2 +	1094	36.59
**Number of military stressors experienced (Wave 2)**		
0	433	17.72
1	730	25.66
2	730	25.55
3	474	17.09
4	262	13.60
5	6	0.37
Mean (SE)	1.84 (0.04)	
**Spouse military service**		
Never	2791	82.87
Former	241	9.41
Current	222	7.71
**Service member paygrade**		
Enlisted	1994	86.84
Warrant or commissioned officer	1263	13.16
**Service member military branch**		
Army	1115	38.43
Navy	607	18.98
Marine Corps	296	13.83
Air Force	1109	23.82
Coast Guard	130	4.94
Other Covariates		
**Time from baseline to follow-up**		
1	649	25.51
2	1486	46.24
3	983	25.96
4	137	2.26
5	2	0.03
Mean (SE)	2.19 (0.80)	

^a^Ns are unweighted and do not consistently add up to 3257 because of missing data. The number missing ranges from 0 (gender and service member characteristics) to 1055 (military stress)

^b^Numbers in parentheses indicate the coding used in regression models

### Readiness outcomes

Spouses had an average of 14.9 lost workdays in the past 3 years. They had an average of 0.6 inpatient days per year, and 9.5 outpatient visit days per year. Spouses’ average score for satisfaction with military support was 1.82. Comparing this score to the coding of the individual items, this falls between ratings of “fair” (1) and “good” (2). The average military stress score was 1.49, which falls between the categories of “slightly stressful” (1) and “moderately stressful” (2).

### Health behaviors and HRQOL

Spouses had an average of 403 combined moderate/vigorous exercise minutes per week. The average ISI score was 7.17, which approximately corresponds to having no clinically significant insomnia. The average number of cigarette packs smoked per day was 0.27, with most spouses (82%) never having smoked or not having smoked in the past year. The average binge drinking frequency in the past year was 1.23, between “never” (1) and “monthly or less” (2). The mean spouse MCS and PCS scores were 49.2 and 53.7, respectively.

### Mediation models

Tables 2 and 3 show results from the mediation models of the five spouse readiness outcomes. Exercise minutes did not have significant direct effects on any of the readiness outcomes. Exercise had negative indirect effects through physical HRQOL (PCS score) on lost workdays (B = -1.40, *p* = 0.015), inpatient days (B = -0.01, *p* = 0.033), and outpatient days (B = -0.17, *p* = 0.009). This indicates that more exercise may be associated with fewer lost workdays, inpatient days, and outpatient days with physical HRQOL as a mediator. However, note that total effects did not reach significance for any outcome.

**Table 2:** Direct and indirect effects of health behaviors and health-related quality of life (HRQOL) on readiness outcomes (*N* = 3,257)

	Lost Workdays					Inpatient Days					Outpatient Days				
	Indir. Eff (MCS12)	Indir. Eff (PCS12)	Total Indirect Eff	Direct Effect	Total Effect	Indir. Eff (MCS12)	Indir. Eff (PCS12)	Total Indirect Effect	Direct Effect	Total Effect	Indir. Eff (MCS12)	Indir. Eff (*P*CS12)	Total Indirect Effect	Direct Effect	Total Effect
Combination exercise minutes	-0.02 (0.21) *p* = 0.945	**-1.40** **(0.58)** ***p* = 0.015**	**-1.42** **(0.57)** ***p* = 0.012**	2.54 (1.75) *p* = 0.146	1.12 (1.73) *p* = 0.516	0.00 (0.00) *P* = 0.937	**-0.01** **(0.01)** ***P* = 0.033**	**-0.01** **(0.01)** ***P* = 0.031**	-0.003 (0.04) *P* = 0.941	-0.01 (0.04) *P* = 0.720	-0.00 (0.05) *P* = 0.938	**-0.17** **(0.07)** ***P* = 0.009**	**-0.18** **(0.07)** ***P* = 0.013**	-0.12 (0.19) *P* = 0.523	-0.30 (0.20) *P* = 0.131
ISI score	**4.91** **(1.25)** ***p* = 0.000**	**5.96** **(1.24)** ***p* = 0.000**	**10.87** **(1.93)** ***p* = 0.000**	-3.41 (2.38) *p* = 0.152	**7.46** **(1.97)** ***p* = 0.000**	0.04 (0.02) *P* = 0.053	**0.04** **(0.013)** ***P* = 0.002**	**0.08** **(0.03)** ***P* = 0.006**	0.01 (0.042) 0.879	**0.09** **(0.036)** ***P* = 0.012**	**1.10** **(0.17)** ***P* = 0.000**	**0.73** **(0.12)** ***P* = 0.000**	**1.83** **(0.22)** ***P* = 0.000**	0.01 (0.30) *P* = 0.987	**1.83** **(0.26)** ***P* = 0.000**
Binge drinking frequency	**0.78** **(0.31)** ***p* = 0.012**	**-1.47** **(0.65)** ***p* = 0.024**	-0.69 (0.62) *p* = 0.265	-2.65 (1.38) *p* = 0.054	**-3.34** **(1.39)** ***p* = 0.016**	0.01 (0.00) *P* = 0.116	**-0.01** **(0.01)** ***P* = 0.039**	-0.00 (0.01) *P* = 0.509	0.05 (0.05) *P* = 0.307	0.05 (0.05) *P* = 0.342	**0.18** **(0.06)** ***P* = 0.007**	**-0.18** **(0.07)** ***P* = 0.014**	-0.00 (0.08) *P* = 0.957	0.22 (0.27) *P* = 0.428	0.21 (0.27) *P* = 0.44
Cigarette packs	0.38 (0.32) *p* = 0.231	1.25 (0.69) *p* = 0.070	**1.64** **(0.66)** ***p* = 0.013**	2.71 (2.78) *p* = 0.333	4.35 (2.90) *p* = 0.133	0.00 (0.00) *P* = 0.308	0.01 (0.01) *P* = 0.107	**0.012** **(0.01)** ***P* = 0.03**	0.03 (0.05) *P* = 0.596	0.04 (0.05) *P* = 0.433	0.09 (0.07) *P* = 0.223	0.15 (0.09) *P* = 0.072	**0.24** **(0.08)** ***P* = 0.005**	**0.72** **(0.32)** ***P* = 0.022**	**0.96** **(0.34)** ***P* = 0.005**
MCS12	N/A	N/A		**-9.44** **(2.46)** ***p* = 0.000**	**-9.44** **(2.46)** ***p* = 0.000**				-0.08 (0.04) *p* = 0.056	-0.08 (0.04) *p* = 0.056				**-2.11** **(0.31)** ***P* = 0.000**	**-2.11** **(0.31)** ***P* = 0.000**
PCS12				**-20.57** **(3.77)** ***p* = 0.000**	**-20.57** **(3.77)** ***p* = 0.000**				**-0.14** **(0.05)** ***P* = 0.001**	**-0.14** **(0.05)** ***P* = 0.001**				**-2.53** **(0.31)** ***P* = 0.000**	**-2.53** **(0.31)** ***P* = 0.000**
Male s*p*ouse	-0.78 (1.02) *p* = 0.443	-0.89 (2.40) *p* = 0.712	-1.67 (2.05) *p* = 0.415	4.55 (8.57) *p* = 0.596	2.88 (8.53) *p* = 0.736	-0.01 (0.01) *p* = 0.479	-0.01 (0.02) *p* = 0.716	-0.01 (0.01) *p* = 0.367	**-0.36 (0.12) *p* = 0.002**	**-0.37 (0.12) *p* = 0.002**	-0.18 (0.23) *p* = 0.442	-0.11 (0.29) *p* = 0.714	-0.28 (0.26) *p* = 0.277	**-4.49 (0.91) *p* = 0.000**	**-4.77 (0.95) *p* = 0.000**
Age: 25–34 years	**Ref**	**Ref**	**Ref**	**Ref**	**Ref**	**Ref**	**Ref**	**Ref**	**Ref**	**Ref**	**Ref**	**Ref**	**Ref**	**Ref**	**Ref**
Age: 17–24 years	-0.99 (0.56) *p* = 0.074	-1.42 (1.22) *p* = 0.243	-2.42 (1.24) *p* = 0.052	-1.92 (2.99) *p* = 0.522	-4.34 (3.16) *p* = 0.170	-0.01 (0.01) *p* = 0.189	-0.01 (0.01) *p* = 0.280	-0.02 (0.01) *p* = 0.085	-0.15 (0.09) *p* = 0.107	-0.17 (0.09) *p* = 0.067	-0.22 (0.12) *p* = 0.067	-0.18 (0.15) *p* = 0.242	**-0.40 (0.17) *p* = 0.019**	-0.48 (0.54) *p* = 0.371	-0.88 (0.56) -*p* = 0.113
Age: 35 + years	1.29 (0.95) *p* = 0.176	**8.49** **(2.73)** ***p* = 0.002**	**9.79** **(2.87)** ***p* = 0.01**	13.71 (8.57) *p* = 0.110	**23.50** **(9.15)** ***p* = 0.010**	0.01 (0.01) *p* = 0.235	**0.06 (0.02) *p* = 0.013**	**0.070 (0.03) *p* = 0.008**	0.12 (0.19) *p* = 0.532	0.19 (0.19) *p* = 0.302	0.29 (0.21) *p* = 0.168	**1.05 (0.30) *p* = 0.000**	**1.33 (0.35) *p* = 0.000**	**1.91 (0.86) *p* = 0.026**	**3.24 (0.80) *p* = 0.000**
White non-His*p*anic	**Ref**	**Ref**	**Ref**	**Ref**	**Ref**	**Ref**	**Ref**	**Ref**	**Ref**	**Ref**	**Ref**	**Ref**	**Ref**	**Ref**	**Ref**
Black non-His*p*anic	0.13 (1.11) *p* = 0.906	3.87 (2.82) *p* = 0.169	4.00 (2.98) *p* = 0.180	**-12.69** **(6.31)** ***p* = 0.044**	-8.69 (5.80) *P* = 0.134	0.00 (0.01) *p* = 0.907	0.03 (0.02) *p* = 0.202	0.03 (0.02) *p* = 0.220	-0.02 (0.11) *p* = 0.835	0.01 (0.11) *p* = 0.965	0.03 (0.25) *p* = 0.905	0.48 (0.34) *p* = 0.166	0.51 (0.42) *p* = 0.225	0.56 (0.88) *p* = 0.520	1.07 (0.88) *p* = 0.226
His*p*anic	0.48 (0.85) *p* = 0.575	-0.98 (1.61) *p* = 0.541	-0.51 (1.63) *p* = 0.756	-3.25 (4.36) *p* = 0.457	-3.757 (4.30) *p* = 0.382	0.00 (0.01) *p* = 0.583	-0.01 (0.01) *p* = 0.550	-0.00 (0.01) *p* = 0.827	**-0.20 (0.07) *p* = 0.007**	**-0.20 (0.07) *p* = 0.005**	0.11 (0.19) *p* = 0.576	-0.12 (0.20) *p* = 0.544	-0.01 (0.24) *p* = 0.954	**-1.13 (0.55) *p* = 0.040**	**-1.14 (0.53) *p* = 0.030**
Other race	**-2.01** **(0.88)** ***p* = 0.017**	**4.47** **(1.91)** ***p* = 0.020**	2.37 (1.82) *p* = 0.192	-7.50 (5.15) *p* = 0.145	-5.13 (4.87) *p* = 0.292	-0.02 (0.01) *p* = 0.109	0.03 (0.02) *p* = 0.056	0.01 (0.02) *p* = 0.451	0.15 (0.17) *p* = 0.365	0.17 (0.17) *p* = 0.319	**-0.47 (0.17) *p* = 0.005**	**0.54 (0.23) *p* = 0.017**	0.07 (0.23) *p* = 0.756	-0.81 (0.70) *p* = 0.247	-0.74 (0.71) *p* = 0.298
Education: Some college/assoc. degree	**Ref**	**Ref**	**Ref**	**Ref**	**Ref**	**Ref**	**Ref**	**Ref**	**Ref**	**Ref**	**Ref**	**Ref**	**Ref**	**Ref**	**Ref**
Education: HS Degree or less	-0.06 (0.66) *p* = 0.924	**3.38** **(1.65)** ***p* = 0.041**	3.31 (1.75) *p* = 0.058	**-9.96** **(4.40)** ***p* = 0.024**	-6.65 (4.21) *p* = 0.114	-0.00 (0.01) *p* = 0.925	0.02 (0.01) *p* = 0.085	0.02 (0.02) *p* = 0.118	-0.08 (0.08) *p* = 0.315	-0.06 (0.09) *p* = 0.474	-0.02 (0.15) *p* = 0.91	**0.41 (0.20) *p* = 0.041**	0.40 (0.25) *p* = 0.105	-0.69 (0.66) *p* = 0.299	-0.29 (0.69) *p* = 0.675
Education: Bachelors degree or higher	0.11 (0.47) *p* = 0.809	-2.30 (1.22) *p* = 0.058	-2.19 (1.22) *p* = 0.072	-2.57 (3.07) *p* = 0.403	-4.76 (3.29) *p* = 0.148	0.00 (0.00) *p* = 0.816	-0.02 (0.01) *p* = 0.079	-0.02 (0.01) *p* = 0.102	-0.09 (0.11) *p* = 0.378	-0.11 (0.11) *p* = 0.308	0.03 (0.11) *p* = 0.814	**-0.29 (0.14) *p* = 0.044**	-0.26 (0.16) *p* = 0.100	-0.23 (0.48) *p* = 0.625	-0.50 (0.49) *p* = 0.313
Married	**Ref**	**Ref**	**Ref**	**Ref**	**Ref**	**Ref**	**Ref**	**Ref**	**Ref**	**Ref**	**Ref**	**Ref**	**Ref**	**Ref**	**Ref**
Divorced/se*p*arated/widowed	2.30 (2.38) *p* = 0.332	-10.40 (6.61) *p* = 0.116	-8.10 (5.38) *p* = 0.132	11.90 (13.44) *p* = 0.376	3.80 (14.84) *p* = 0.798	0.02 (0.02) *p* = 0.371	-0.07 (0.05) *p* = 0.141	-0.05 (0.04) *p* = 0.178	-0.22 (0.16) *p* = 0.162	**-0.27 (0.14) *p* = 0.047**	0.52 (0.52) *p* = 0.319	-1.28 (0.79) *p* = 0.108	-0.76 (0.59) *p* = 0.194	-0.81 (1.46) *p* = 0.578	-1.57 (1.71) *p* = 0.358
No. of children: 0	**Ref**	**Ref**	**Ref**	**Ref**	**Ref**	**Ref**	**Ref**	**Ref**	**Ref**	**Ref**	**Ref**	**Ref**	**Ref**	**Ref**	**Ref**
No. of children: 1	0.22 (0.57) *p* = 0.696	-0.46 (1.31) *p* = 0.725	-0.24 (1.23) *p* = 0.846	-3.18 (3.70) *p* = 0.390	-3.42 (3.89) = 0.380	0.00 (0.01) *p* = 0.708	-0.00 (0.01) *p* = 0.718	-0.00 (0.01) *p* = 0.876	-0.02 (0.10) 0.837	-0.02 (0.10) *p* = 0.827	0.05 (0.13) *p* = 0.697	-0.06 (0.16) *p* = 0.718	-0.01 (0.16) *p* = 0.959	-0.05 (0.54) *p* = 0.920	-0.06 (0.56) *p* = 0.912
No. of children: 2 +	-0.03 (0.54) *p* = 0.964	-0.97 (1.25) *p* = 0.435	-1.00 (1.22) *p* = 0.414	-2.27 (3.41) *p* = 0.505	-3.27 (3.49) *p* = 0.348	0.00 (0.01) *p* = 0.956	-0.01 (0.01) *p* = 0.415	-0.01 (0.01) *p* = 0.399	**-0.37 (0.09) *p* = 0.000**	**-0.38 (0.09) *p* = 0.000**	-0.01 (0.12) *p* = 0.961	-0.12 (0.15) *p* = 0.412	-0.13 (0.17) *p* = 0.435	**-1.55 (0.55) *p* = 0.005**	**-1.68 (0.56) *p* = 0.003**
Officer	-0.84 (0.47) p = 0.075	**-2.58** **(1.12)** **p = 0.023**	**-3.42** **(1.18)** **p = 0.004**	0.31 (2.43) p = 0.897	-3.10 (2.51) p = 0.215	-0.01 (0.01) p = 0.161	**-0.02 (0.01) p = 0.046**	**-0.03 (0.01) p = 0.020**	0.07 (0.07) p = 0.312	0.05 (0.07) p = 0.502	-0.19 (0.10) p = 0.059	**-0.32 (0.13) p = 0.015**	**-0.50 (0.15) p = 0.001**	-0.44 (0.40) p = 0.270	**-0.94 (0.41) p = 0.023**
Army	**Ref**	**Ref**	**Ref**	**Ref**	**Ref**	**Ref**	**Ref**	**Ref**	**Ref**	**Ref**	**Ref**	**Ref**	**Ref**	**Ref**	**Ref**
Navy	-0.53 (0.58) p = 0.366	-2.49 (1.47) p = 0.090	**-3.02** **(1.45)** **p = 0.038**	1.55 (3.63) p = 0.670	-1.47 (3.66) P = 0.687	-0.01 (0.01) p = 0.401	-0.02 (0.01) p = 0.112	-0.02 (0.01) p = 0.053	0.02 (0.10) p = 0.863	-0.01 (0.10) p = 0.964	-0.12 (0.13) p = 0.367	-0.31 (0.17) p = 0.074	**-0.43 (0.19) p = 0.023**	-0.24 (0.51) p = 0.637	-0.67 (0.53) p = 0.210
Marine Corps	0.46 (0.74) p = 0.536	-1.14 (1.72) *p* = 0.507	-0.68 (1.75) *p* = 0.696	3.27 (5.79) *p* = 0.572	2.59 (5.99) *p* = 0.666	0.00 (0.01) *p* = 0.566	-0.01 (0.01) *p* = 0.507	-0.00 (0.01) *p* = 0.758	-0.01 (0.13) *p* = 0.969	-0.01 (0.13) *p* = 0.945	0.103 (0.17) *p* = 0.535	-0.14 (0.21) *p* = 0.506	-0.04 (0.24) *p* = 0.876	1.13 (0.77) *p* = 0.143	1.10 (0.80) *p* = 0.172
Air Force	0.20 (0.54) *p* = 0.710	-2.25 (1.32) *p* = 0.087	-2.05 (1.28) *p* = 0.110	0.99 (3.17) *p* = 0.755	-1.06 (3.21) *p* = 0.740	0.00 (0.01) *p* = 0.714	-0.02 (0.01) *p* = 0.110	-0.01 (0.01) *p* = 0.146	-0.09 (0.08) *p* = 0.277	-0.10 (0.08) *p* = 0.208	0.05 (0.12) *p* = 0.706	-0.28 (0.16) *p* = 0.079	-0.231 (0.17) *p* = 0.174	0.21 (0.54) *p* = 0.694	-0.02 (0.53) *p* = 0.970
Coast Guard	-0.54 (0.99) *p* = 0.582	-1.34 (1.94) *p* = 0.489	-1.89 (1.94) *p* = 0.331	-5.99 (3.80) *p* = 0.115	**-7.88** **(3.73)** ***p* = 0.035**	-0.01 0.01 *p* = 0.596	-0.01 (0.01) *p* = 0.493	-0.01 (0.01) *p* = 0.326	-0.13 (0.11) *p* = 0.244	-0.14 (0.12) *p* = 0.196	-0.12 (0.22) *p* = 0.582	-0.16 (0.24) *p* = 0.488	-0.29 (0.28) *p* = 0.305	-0.26 (0.84) *p* = 0.754	-0.55 (0.92) *p* = 0.552
S*p*ouse Military Service: Never	**Ref**	**Ref**	**Ref**	**Ref**	**Ref**	**Ref**	**Ref**	**Ref**	**Ref**	**Ref**	**Ref**	**Ref**	**Ref**	**Ref**	**Ref**
S*p*ouse Military Service: Current	-0.75 (0.97) *p* = 0.436	1.51 (2.23) *p* = 0.496	0.76 (1.92) *p* = 0.692	-8.56 (5.56) *p* = 0.123	-7.80 (5.67) *p* = 0.169	-0.01 (0.01) *p* = 0.457	0.01 (0.02) *p* = 0.500	0.00 (0.01) *p* = 0.770	-0.13 (0.12) *p* = 0.261	-0.13 (0.12) *p* = 0.276	-0.17 (0.21) *p* = 0.423	0.19 (0.27) *p* = 0.495	0.02 (0.24) *p* = 0.947	**3.80 (1.00) *p* = 0.000**	**3.81 (1.01) *p* = 0.000**
S*p*ouse Military Service: Former	-0.19 (0.95) *p* = 0.839	**8.16** **(2.86)** ***p* = 0.004**	**7.96** **(2.77)** ***p* = 0.004**	-7.61 (6.76) *p* = 0.260	0.36 (6.73) *p* = 0.958	-0.00 (0.01) *p* = 0.839	**0.06 (0.02) *p* = 0.019**	**0.06 (0.02) *p* = 0.017**	**-0.21 (0.10) *p* = 0.032**	-0.15 (0.09) *p* = 0.105	-0.04 (0.21) *p* = 0.838	**1.00 (0.32) *p* = 0.002**	**0.96 (0.32) *p* = 0.003**	**-2.41 (0.84) *p* = 0.004**	-1.45 (0.84) *p* = 0.083
Time between survey waves	0.15 (0.21) *p* = 0.487	**-0.94** **(0.465)** ***p* = 0.044**	-0.79 (0.47) *p* = 0.094	**2.62** **(1.28)** ***p* = 0.040**	1.83 (1.21) *p* = 0.132	0.00 (0.00) *p* = 0.521	-0.01 (0.00) *p* = 0.056	-0.01 (0.00) *p* = 0.135	0.04 (0.04) *p* = 0.213	0.04 (0.04) *p* = 0.271	0.03 (0.05) *p* = 0.486	**-0.12 (0.06) *p* = 0.035**	-0.08 (0.07) *p* = 0.199	0.04 (0.21) *p* = 0.841	-0.04 (0.22) *p* = 0.847

^*^Note: Models are mediation models with survey weights a*pp*lied. Model coefficients re*p*orted with standard errors in *p*arentheses. All health behaviors, MCS12, PCS12, and time between survey waves are standardized variables with mean = 0 and standard deviation = 1 (z-scores). All models adjusted for spouse demogra*p*hics and military characteristics. Significant results at the *p*<0.05 level are bolded

**Table 3:** Direct and indirect effects of health behaviors and health-related quality of life (HRQOL) on readiness outcomes (*N* = 3,257)

	Military Satisfaction (z-score)					Military Stress (z-score)				
	Indir. Eff (MCS12)	Indir. Eff (PCS12)	Total Indirect Eff	Direct	Total Effect	Indir. Eff (MCS12)	Indir. Eff (PCS12)	Total Indirect Effect	Direct Effect	Total Effect
Combination exercise minutes	0.00 (0.00) *P* = 0.939	0.01 (0.00) *P* = 0.100	0.01 (0.01) *P* = 0.297	-0.03 (0.03) *P* = 0.296	-0.02 (0.03) *P *= 0.401	0.00 (0.00) *P* = 0.945	0.00 (0.00) *P* = 0.604	0.00 (0.01) *P* = 0.857	-0.03 (0.03) *P *= 0.382	-0.03 (0.03) *P* = 0.382
ISI score	**-0.10** **(0.02)** ***P *= 0.000**	**-0.02** **(0.01)** ***P* = 0.042**	**-0.12** **(0.02)** ***P* = 0.000**	**-0.10** **(0.04)** ***P* = 0.012**	**-0.22** **(0.03)** ***P* = 0.000**	**0.09** **(0.02)** ***P* = 0.000**	-0.01 (0.01) *P* = 0.594	**0.09** **(0.03)** ***P* = 0.001**	**0.09** **(0.04)** ***P* = 0.028**	**0.18** **(0.03)** ***P* = 0.000**
Binge drinking frequency	**-0.02** **(0.01)** ***P* = 0.011**	0.01 (0.00) *P* = 0.114	-0.01 (0.01) *P* = 0.066	0.03 (0.03) *P* = 0.342	0.02 (0.03) *P* = 0.589	**0.02** **(0.01)** ***P* = 0.015**	0.00 (0.00) *P* = 0.604	**0.02** **(0.01)** ***P* = 0.009**	-0.01 (0.03) *P* = 0.878	0.01 (0.04) *P* = 0.760
Cigarette packs	-0.01 (0.01) *P* = 0.209	-0.00 (0.00) *P* = 0.148	-0.01 (0.01) *P* = 0.051	-0.03 (0.03) *P* = 0.329	-0.04 (0.03) *P* = 0.180	0.01 (0.01) *P* = 0.199	-0.00 (0.00) *P* = 0.605	0.01 (0.01) *P* = 0.314	0.02 (0.05) *P* = 0.607	0.03 (0.05) *P* = 0.514
MCS12				**0.19** **(0.04)** ***P* = 0.000**	**0.19** **(0.04)** ***P* = 0.000**				**-0.18** **(0.04)** ***P* = 0.000**	**-0.18** **(0.04)** ***P* = 0.000**
PCS12				**0.07** **(0.03)** ***P* = 0.037**	**0.07** **(0.03)** ***P* = 0.037**				0.02 (0.03) *P* = 0.596	0.02 (0.03) *P* = 0.596
Male spouse	0.02 (0.02) *p* = 0.448	0.00 0.01 *p* = 0.714	0.02 (0.02) *p* = 0.306	-0.03 (0.14) *p* = 0.854	-0.01 (0.14) *p* = 0.963	-0.02 (0.02) *P* = 0.450	0.00 (0.00) *P* = 0.750	-0.01 (0.02) *P* = 0.496	-0.20 (0.13) *P* = 0.125	-0.21 (0.13) *P* = 0.109
Age: 25–34	**Ref**	**Ref**	**Ref**	**Ref**	**Ref**	**Ref**	**Ref**	**Ref**	**Ref**	**Ref**
Age: 17–24 years	0.02 (0.01) *p* = 0.062	0.01 (0.01) *p* = 0.318	**0.03 (0.01) *p* = 0.027**	**-0.23 (0.07) *p* = 0.001**	**-0.20 (0.07) *p* = 0.004**	-0.02 (0.01) *P* = 0.079	0.00 (0.00) *P* = 0.605	-0.02 (0.01) *P* = 0.124	**0.20** **(0.08)** ***P* = 0.012**	**0.18** **(0.08)** ***P* = 0.024**
Age: 35 + years	-0.03 (0.02) *P* = 0.199	-0.03 (0.02) *p* = 0.061	**-0.05 (0.03) *p* = 0.038**	-0.10 (0.11) *p* = 0.355	-0.16 (0.12) *p* = 0.176	0.02 (0.02) *P* = 0.212	-0.01 (0.01) *P* = 0.597	0.02 (0.03) *P* = 0.489	0.11 (0.11) *P* = 0.303	0.13 (0.12) *P* = 0.266
White, non-Hispanic	**Ref**	**Ref**	**Ref**	**Ref**	**Ref**	**Ref**	**Ref**	**Ref**	**Ref**	**Ref**
Black, non-Hispanic	-0.00 (0.02) *p* = 0.904	-0.01 (0.01) *p* = 0.229	-0.02 (0.03) *p* = 0.535	**0.28 (0.13) *p* = 0.034**	**0.26 (0.13) *p* = 0.043**	0.00 (0.02) *P* = 0.928	-0.00 (0.00) *P* = 0.627	-0.00 (0.02) *P* = 0.955	-0.14 (0.14) *P* = 0.315	-0.14 (0.14) *P* = 0.318
His*p*anic	-0.01 (0.02) *p* = 0.578	0.00 (0.01) *p* = 0.550	-0.01 (0.02) *p* = 0.700	-0.04 (0.10) *p* = 0.705	-0.04 (0.10) *p* = 0.670	0.01 (0.02) *P* = 0.610	0.00 (0.00) *P* = 0.715	0.01 (0.02) *P* = 0.586	-0.14 (0.10) *P* = 0.174	-0.13 (0.10) *P* = 0.219
Other race	**0.04 (0.02) *p* = 0.008**	-0.01 (0.01) *p* = 0.091	0.03 (0.02) *p* = 0.079	**0.30 (0.11) *p* = 0.005**	**0.33 (0.11) *p* = 0.003**	**-0.04** **(0.02)** ***P* = 0.009**	-0.00 (0.01) *P* = 0.611	**-0.04** **(0.02)** ***P* = 0.007**	-0.10 (0.10) *P* = 0.341	**-0.14** **(0.11)** ***P* = 0.007**
Education: Some college/assoc. degree	**Ref**	**Ref**	**Ref**	**Ref**	**Ref**	**Ref**	**Ref**	**Ref**	**Ref**	**Ref**
Education: HS Degree or less	0.00 (0.01) *p* = 0.920	-0.01 (0.01) *p* = 0.135	-0.01 (0.02) *p* = 0.518	0.01 (0.09) *p* = 0.959	-0.01 (0.10) *p* = 0.959	-0.00 (0.01) *P* = 0.936	-0.00 (0.01) *P* = 0.607	-0.00 (0.01) *P* = 0.783	-0.08 (0.11) *P* = 0.478	-0.08 (0.11) *P* = 0.462
Education: Bachelors degree or higher	-0.00 (0.01) *P* = 0.818	0.01 (0.01) *p* = 0.154	0.01 (0.01) *p* = 0.607	**-0.14 (0.07) *p* = 0.035**	**-0.13 (0.07) *p* = 0.043**	0.00 (0.01) *P* = 0.792	0.00 (0.00) *P* = 0.605	0.00 (0.01) *P* = 0.671	**0.17** **(0.07)** ***P* = 0.016**	**0.176** **(0.07)** ***P* = 0.014**
Married	**Ref**	**Ref**	**Ref**	**Ref**	**Ref**	**Ref**	**Ref**	**Ref**	**Ref**	**Ref**
Divorced/separated/widowed	-0.05 (0.05) *p* = 0.321	0.03 (0.03) *p* = 0.197	-0.01 (0.04) *p* = 0.739	-0.60 (0.38) *p* = 0.110	-0.62 (0.40) *p* = 0.119	0.04 (0.04) *P* = 0.339	0.01 (0.02) *P* = 0.615	0.05 (0.05) *P* = 0.309	0.38 (0.314) *P* = 0.228	0.43 (0.32) *P* = 0.183
No. of children: 0	**Ref**	**Ref**	**Ref**	**Ref**	**Ref**	**Ref**	**Ref**	**Ref**	**Ref**	**Ref**
No. of children: 1	-0.01 (0.01) *p* = 0.694	0.00 (0.00) *p* = 0.729	-0.00 (0.01) *p* = 0.781	0.02 (0.07) *p* = 0.789	0.02 (0.07) *p* = 0.827	0.00 (0.01) *P* = 0.691	0.00 (0.00) *P* = 0.764	0.005 (0.011) *P* = 0.676	**0.19** **(0.08)** ***P* = 0.017**	**0.20** **(0.08)** ***P* = 0.016**
No. of children: 2 +	0.00 (0.01) *p* = 0.960	0.00 (0.00) *p* = 0.447	0.00 (0.01) *p* = 0.720	-0.06 (0.07) *p* = 0.411	-0.05 (0.07) *p* = 0.452	-0.00 (0.01) *P* = 0.961	0.00 (0.00) *P* = 0.660	0.00 (0.01) *P* = 0.977	**0.215** **(0.07)** ***P* = 0.003**	**0.22** **(0.07)** ***P* = 0.004**
Officer	0.02 (0.01) *p* = 0.062	0.01 (0.01) *p* = 0.110	**0.03 (0.01) *p* = 0.011**	-0.07 (0.06) *p* = 0.268	-0.04 (0.06) *p* = 0.494	-0.02 (0.01) *P* = 0.069	0.00 (0.00) *P* = 0.603	-0.014 (0.01) *P* = 0.172	0.112 (0.07) *P* = 0.085	0.10 (0.07) *P* = 0.133
Army	**Ref**	**Ref**	**Ref**	**Ref**	**Ref**	**Ref**	**Ref**	**Ref**	**Ref**	**Ref**
Navy	0.01 (0.01) *p* = 0.359	0.01 (0.01) *p* = 0.167	0.02 (0.01) *p* = 0.106	0.08 (0.08) *p* = 0.319	0.10 (0.08) *p* = 0.227	-0.01 (0.01) *P* = 0.427	0.00 (0.00) *P* = 0.602	-0.01 (0.01) *P* = 0.582	0.10 (0.08) *P* = 0.222	0.09 (0.08) *P* = 0.263
Marine Corps	-0.01 (0.02 *p* = 0.536	0.00 (0.01) *p* = 0.519	-0.01 (0.02) *p* = 0.711	0.06 (0.10) *p* = 0.513	0.06 (0.10) *p* = 0.561	0.01 (0.01) *P* = 0.529	0.00 (0.00) *P* = 0.680	0.01 (0.01) *P* = 0.500	-0.00 (0.10) *P* = 0.989	0.01 (0.10) *P* = 0.933
Air Force	-0.00 (0.01) *p* = 0.708	0.01 (0.01) *p* = 0.151	0.03 (0.01) *p* = 0.769	0.14 (0.07) *p* = 0.053	0.14 (0.07) *p* = 0.050	0.01 (0.01) *P* = 0.655	0.00 (0.00) *P* = 0.608	0.01 (0.01) *P* = 0.566	0.06 (0.08) *P* = 0.460	0.06 (0.08) *P* = 0.419
Coast Guard	0.01 (0.02) *p* = 0.581	0.00 (0.01) *p* = 0.508	0.02 (0.02) *p* = 0.430	0.06 (0.12) *p* = 0.608	0.08 (0.12) *p* = 0.520	-0.01 (0.02) *P* = 0.608	0.00 (0.00) *P* = 0.663	-0.01 (0.02) *P* = 0.660	-0.33 (0.19) *P* = 0.081	-0.34 (0.18) *P* = 0.068
S*p*ouse Military Service: Never	**Ref**	**Ref**	**Ref**	**Ref**	**Ref**	**Ref**	**Ref**	**Ref**	**Ref**	**Ref**
S*p*ouse Military Service: Current	0.02 (0.02) *p* = 0.436	-0.01 (0.01) *p* = 0.504	0.01 (0.02) *p* = 0.548	-0.05 (0.15) *p* = 0.731	-0.04 (0.16) *p* = 0.787	-0.01 (0.02) *P* = 0.424	-0.00 (0.00) *P* = 0.657	-0.02 (0.02) *P* = 0.408	0.17 (0.136) *P* = 0.210	0.15 (0.14) *P* = 0.266
S*p*ouse Military Service: Former	0.00 (0.02) *p* = 0.838	-0.03 (0.02) *p* = 0.08	-0.02 (0.02) *p* = 0.316	-0.13 (0.11) *p* = 0.222	-0.15 (0.11) *p* = 0.156	-0.00 (0.02) *P* = 0.830	-0.01 (0.01) *P* = 0.600	-0.01 (0.02) *P* = 0.651	0.03 (0.11) *P* = 0.774	0.02 (0.11) *P* = 0.848
No. of military-related stressors						0.00 (0.01) *P* = 0.619	0.00 (0.00) *P* = 0.707	0.00 (0.01) *P* = 0.592	**0.12** **(0.04)** ***P* = 0.003**	**0.12** **(0.04)** ***P* = 0.003**
Time between survey waves	-0.00 (0.00) *p* = 0.489	0.00 (0.00) *p* = 0.116	0.00 (0.00) *p* = 0.982	0.02 (0.03) *p* = 0.433	0.02 (0.03) *p* = 0.447	0.00 (0.00) *P* = 0.502	0.00 (0.00) *P* = 0.611	0.00 (0.00) *P* = 0.420	-0.00 (0.03) *P* = 0.942	0.00 (0.03) *P* = 0.968

^*^Note: Models are mediation models with survey weights applied. Model coefficients reported with standard errors in parentheses. All health behaviors, MCS12, PCS12, number of military stressors and time between survey waves are standardized variables with mean = 0 and standard deviation = 1 (z-scores). All models adjusted for spouse demographics and military characteristics. ﻿Significant results at the *p*<0.05 level are bolded

Insomnia symptoms, as measured by the ISI score, had a negative direct effect on satisfaction with military support (B = -0.10, *p* = 0.012) and a positive direct effect on military stress (B = 0.09, *p *= 0.028). Insomnia also had significant indirect effects on all five readiness outcomes, via physical and/or mental HRQOL. With the MCS score as a mediator, insomnia had positive indirect effects on lost workdays (B = 4.91, *p* < 0.001), outpatient days (B = 1.10, *p* < 0.001), and military stress (B = 0.09, *p* < 0.001), and a negative indirect effect on satisfaction with military support (B = -0.10, *p* < 0.001). With the PCS score as a mediator, insomnia had positive indirect effects on lost workdays (B = 5.96, *p* < 0.001), inpatient days (B = 0.04, *p* = 0.002), outpatient days (B = 0.73, *p* < 0.001), and a negative indirect effect on satisfaction with military support (B = -0.02, *p* = 0.042). This indicates that greater insomnia symptoms, working through HRQOL as a mediator, were associated with more lost workdays, inpatient days, and outpatient days, as well as greater military stress, and lower satisfaction with military support. Finally, insomnia was the only health behavior with significant total effects for all five spouse health readiness outcomes.

There were no direct effects of binge drinking frequency on readiness outcomes. However, binge drinking did have indirect effects on all five readiness measures. With the MCS score as a mediator, binge drinking had positive indirect effects on lost workdays (B = 0.78, *p* = 0.012), outpatient days (B = 0.18, *p* = 0.007), and military stress (B = 0.02, p = 0.016), and a negative indirect effect on satisfaction with military support (B = -0.02, *p* = 0.011). With the PCS score as a mediator, unexpectedly, binge drinking had negative indirect effects on lost workdays (B = -1.47, *p* = 0.024), inpatient days (B = -0.01, *p* = 0.039) and outpatient days (B = -0.18, *p* = 0.014). Also, since the direction of the effects through the MCS and PCS diverged for some outcomes, the total effect of binge drinking was only significant for lost workdays (see Tables 2 and 3).

Smoking, as measured by the number of cigarette packs smoked per day, had both direct and indirect effects on lost workdays, inpatient days, and outpatient days. There was a positive direct effect of smoking on outpatient days (B = 0.72, *p* = 0.022). While the individual path effects through the MCS and PCS scores separately were not statistically significant, there were significant total positive indirect effects on lost workdays (B = 1.64, *p* = 0.013), inpatient days (B = 0.012, *p* = 0.030), and outpatient days (B = 0.24, *p* = 0.005). This indicates that working through HRQOL, smoking was associated with more lost workdays, inpatient days, and outpatient days. There was also a significant total effect of smoking on outpatient days (B = 0.96, *p* = 0.005).

All readiness outcomes were significantly predicted by a at least one HRQOL indicator, and both mental and physical HRQOL had direct effects on three of five outcomes (lost workdays, outpatient days, and satisfaction with military support) The MCS score had a negative direct effect on lost workdays (B = -9.44, *p* < 0.001), outpatient days (B = -2.11, *p* < 0.001), and military stress (B = -0.18, *p* < 0.001), such that higher mental health functioning was associated with fewer lost workdays, outpatient days, and less military stress. Higher mental health functioning was directly associated with greater satisfaction with military support (B = 0.19, *p* < 0.001). The PCS score had a negative direct effect on lost workdays (B = -20.57, *p* < 0.001), inpatient days (B = -0.14, *p* = 0.001), outpatient days (B = -2.53, *p* < 0.001). Finally, higher physical health functioning was directly associated with greater satisfaction with military support (B = 0.07, *p* = 0.037).

## Discussion

This study utilized longitudinal data from the Millennium Cohort Family Study to assess the relationship of health behaviors and HRQOL with spouse readiness outcomes among over 3,000 military spouses. The study sought to identify health behaviors and HRQOL indices that may enable the DHA and military communities to better support and prepare spouses for the evolving demands of military-connected life. Results indicated that better mental and physical HRQOL was significantly associated with higher levels of spouse readiness as defined by fewer lost workdays, fewer outpatient visits, and higher military satisfaction. Mental and physical HRQOL also fully or partially mediated the effects of focal health behaviors on readiness outcomes. Altogether, the results demonstrate that health behaviors and mental and physical HRQOL account for meaningful variation in relation to spouse readiness outcomes and that HRQOL serves as a useful composite index to capture most impacts of the focal health behaviors we assessed on readiness. Therefore, routine assessment and surveillance of HRQOL among military spouses may prove useful to health care and service providers as a gross indicator of population readiness and may suggest potential modifiable health vulnerabilities and protective factors that could be targeted to bolster spouse readiness.

We noted unique patterns of association with spouse readiness for each of the four health behaviors included as predictors in our analyses. However, experiencing more insomnia symptoms was the only outcome consistently associated with poorer readiness across outcomes, and effects were only partially mediated by physical and mental HRQOL. This is likely due to the pervasive effects of poor sleep on a range of health outcomes that may hinder readiness, including poorer physical and mental health [53, 54]. Smoking also was directly and indirectly associated with more outpatient healthcare visits. Exercise, though, ultimately did not have any significant total effects on any readiness outcomes, although the pattern of our results suggested that engaging in more frequent exercise in a typical week may be indirectly associated with fewer inpatient and outpatient health care services and fewer lost workdays, mediated by physical HRQOL. Binge drinking was indirectly associated with readiness, but unexpectedly had competing positive (e.g., decreased hospitalization days) and negative (e.g., increased military stress, decreased satisfaction with military support, increased lost workdays) effects. The finding that binge drinking was associated with decreased inpatient days, outpatient days, and lost workdays via physical HRQOL may be in part due to higher alcohol use among younger spouses who are generally healthier than older spouses. Our relatively healthy and young military spouse population also may account for the somewhat weaker associations noted between smoking, HRQOL and readiness outcomes.

The Department of Defense has invested in promoting spouse readiness and resilience to enhance mission readiness and the health of the military force [55]. Several resources and programs have been developed focusing on children and the family unit, many of which are designed to support families during or after stressful exposures such as deployment to prevent negative family outcomes (e.g., [56–58]). These programs have proven essential in extending support networks for families and improving family functioning during particularly challenging periods (e.g., overseas assignment, deployment). However, it can be difficult for spouses to navigate and identify these resources when needed due to the de-centralization and at times duplication of services across military and community settings [55]. Military OneSource offers a cohesive military spouse readiness support system to active duty and reserve component families, providing a host of coordinated resources to help families such as deployment assistance, financial readiness, violence prevention and response services, and transition assistance among others.

Results from the 2021 Military Family Lifestyle Survey suggest that military families are in even greater need of support and resiliency-enhancing services given the increased demands on and stress among military families in recent years (e.g., related to COVID-19 and government unrest) [59]. In fact, 24% of active-duty family respondents reported that military quality of life was a top concern due to the instability and inconsistency of daily life (e.g., time with family/children, difficulty of relocating, operational tempo, time away). Approximately 7% indicated that quality of life challenges were related to accessing needed resources and supports. Fewer than half of spouses rated their health as excellent. Population level screening results for HRQOL could inform how integrated sources of information for military families, such as Military OneSource, better target families at risk and promote family readiness.

Research demonstrates that some military spouses may be at greater risk for lower resilience and poorer health-related quality of life, such as those in primary caregiving roles for injured partners [60]. Injuries incurred during deployment among service members are also associated with poorer long-term health-related quality of life, which could impact the well-being of dual military spouses who experience an injury in service [61]. Future research should assess the relationship of quality of life and resilience among these and other military spouse subgroups, to determine if risk factors vary so that support services can be further tailored to meet specific needs.

### Strengths and Limitations

The study has several limitations. First, in these analyses we focused on spouse readiness related to spousal health behaviors (e.g., lost workdays, health care seeking), military-related stress, and perceived military support. This focus was selected at the request of the DHA, to support future improvements in surveillance of health factors associated with readiness. We did not include a focus on service member readiness, which is addressed in a separate manuscript [62]. Also, we did not evaluate variables focused on children’s well-being, and this should be a focus for future research. The sample further was restricted to heterosexual married couples; therefore, findings do not generalize to same-sex or unmarried, cohabitating military couples. In addition, the independent variables and mediators were assessed at the same time point (Wave 1), rather than at different times. Also, the outcome lost workdays was assessed at follow-up with a reference period of the past three years, while the time between survey waves for some participants was less than three years. This means that for some respondents, some portion of estimated lost workdays may precede the Wave 1 interview date. In addition, some independent variables had skewed distributions, due to a small number of respondents at higher levels of those measures. This likely resulted in conservative estimates of those relationships. Finally, the data were collected in years 2011–2013 (wave 1) and 2014–2016 (wave 2), which may affect generalizability to present day military spouses. Strengths of the study include the longitudinal design, inclusion of military spouses across all military branches, and the use of standardized scales to assess key predictors of interest including health-related quality of life and health behaviors.

## Conclusions

This study has the potential to contribute to the military health system meeting its quadruple aim (i.e., increased readiness, better health, better care, and lower cost), given the established importance of spouse satisfaction and support for service member readiness [13, 14] and the long-term health and economic costs of poor health behaviors [29]. We identified two measurable and modifiable health behaviors directly related to spouse readiness, including insomnia, and smoking. Insomnia, in particular, had consistently significant and negative impacts on all readiness outcomes. Results revealed that mental and physical HRQOL partially mediated behavioral effects for insomnia and smoking and fully mediated any potential effects of exercise on spouse readiness. These findings suggest that consistently assessing and addressing HRQOL in military spouse healthcare settings (e.g., primary care), as well as targeting specific health behaviors such as insomnia through family support services may help prevent poorer outcomes during times of heightened stress for military families (e.g., family separation, deployment) and enhance spouse resiliency. HRQOL may also be utilized as a brief screening tool, in concert with more comprehensive assessment of family well-being, to assess readiness to serve among military families. The DHA has efforts underway to systematically monitor HRQOL, in order to better address threats to population-level readiness. These findings will help focus that assessment effort.

## Data Availability

The data that support the findings of this study are not publicly available, but can be requested for professional use from the Center for Deployment Health, Naval Health Research center (https://www.familycohort.org; usn.point-loma.navhlthrschcensan.mbx.family-cohort@health.mil); restrictions apply to the use of these data, which were accessed under contract for the current study.
